# COL10A1 allows stratification of invasiveness of colon cancer and associates to extracellular matrix and immune cell enrichment in the tumor parenchyma

**DOI:** 10.3389/fonc.2022.1007514

**Published:** 2022-10-04

**Authors:** Ulf D. Kahlert, Wenjie Shi, Marco Strecker, Lorenz A. Scherpinski, Thomas Wartmann, Maximilian Dölling, Aristotelis Perrakis, Borna Relja, Miriam Mengoni, Andreas Braun, Roland S. Croner

**Affiliations:** ^1^ University Clinic for General, Visceral, Vascular and Transplantation Surgery, Faculty of Medicine, Otto-von-Guericke-University, Magdeburg, Germany; ^2^ University Hospital for Gynecology, Pius-Hospital, University Medicine Oldenburg, Oldenburg, Germany; ^3^ Experimental Radiology, University Clinic of Radiology and Nuclear Medicine, Faculty of Medicine, Otto-von-Guericke-University, Magdeburg, Germany; ^4^ University Clinic for Dermatology, Faculty of Medicine, Otto-von-Guericke-University, Magdeburg, Germany

**Keywords:** colon cancer, biomarker, tumor microenvironment, collagen type 10, prognosis

## Abstract

**Background:**

Treatment options for metastatic colorectal cancer (CRC) are mostly ineffective. We present new evidence that tumor tissue collagen type X alpha 1 (COL10A1) is a relevant candidate biomarker to improve this dilemma.

**Methods:**

Several public databases had been screened to observe COL10A1 expression in transcriptome levels with cell lines and tissues. Protein interactions and alignment to changes in clinical parameters and immune cell invasion were performed, too. We also used algorithms to build a novel COL10A1-related immunomodulator signature. Various wet-lab experiments were conducted to quantify COL10A1 protein and transcript expression levels in disease and control cell models.

**Results:**

COL10A1 mRNA levels in tumor material is clinical and molecular prognostic, featuring upregulation compared to non-cancer tissue, increase with histomorphological malignancy grading of the tumor, elevation in tumors that invade perineural areas, or lymph node invasion. Transcriptomic alignment noted a strong positive correlation of COL10A1 with transcriptomic signature of cancer-associated fibroblasts (CAFs) and populations of the immune compartment, namely, B cells and macrophages. We verified those findings in functional assays showing that COL10A1 are decreased in CRC cells compared to fibroblasts, with strongest signal in the cell supernatant of the cells.

**Conclusion:**

COL10A1 abundance in CRC tissue predicts metastatic and immunogenic properties of the disease. COL10A1 transcription may mediate tumor cell interaction with its stromal microenvironment.

## Introduction

Colorectal cancer remains to be one of the most malignant and deadliest cancers worldwide, with over 935 thousand deaths and more than 1.9 million new cases in 2020 (1), despite progressive scientific efforts. Peritoneal and hepatic distant metastases barely provide a median survival rate of 5–9 months upon diagnostic detection (2, 3). Current clinical diagnostics to appreciate the tumor location and spread involve digital rectal examination (DRE), total colposcopy with biopsy, abdominal sonography, thoracal X-ray, carcinoembryonic antigen (CEA) blood levels, and abdominal/thoracal computer tomography, and for rectum carcinoma, rigid rectoscopy, pelvic MR/CT, and rectal end sonography in case of locally limited tumors. One of the major deficiencies in the staging diagnosis of CRC is the detection of malignant lymph nodes and stratification of tumor cases with elevated metastatic risk in low and medium malignancy staging (II/III) (4) (5). On the one hand, this is due to inconsistent cutoff limits and on the other hand to poor sensitivity and specificity of conventional CT (70%, 78%) (6) (71%, 67%) (7) or CT colonography (CTC) (<70%) (8) detecting not only metastatic enlarged but also micro-metastatic lymph nodes with normal size, urging the need for improved diagnostics such as MDCT. Another way is to improve the diagnostic criteria for CT-diagnosed lymph node changes. As a result, based on recent consensus data, the largest short diameter of the suspicious tissue and internal heterogeneity have been identified as the best criteria for CT-assisted malignancy detection (9). Poor diagnosis is particularly problematic, as lymph node status determines whether adjuvant chemotherapy is indicated or not. Colon carcinomas are treated with adjuvant chemotherapy from the Union for International Cancer Control (UICC) stage II/III and rectum carcinoma depending on locality from UICC II in the middle and lower rectum with neoadjuvant regime (upper rectum with adjuvant chemotherapy) (10). Currently, neoadjuvant chemotherapy is not considered the standard of care for CRC patients; however, recent data indicate significant advantages when applying pre-surgical chemotherapy over conventional adjuvant chemotherapy in terms of OS and DSS (11). Contrary to adjuvant chemotherapy, which is started after the pathological evaluation of the resected lymph nodes, initiation and monitoring of neoadjuvant chemotherapy have so far mostly relied on imaging parameters of the tumor area alone. Oncologist and radiologist are frequently confronted with the dilemma of the inability to unequivocally discriminate false positivity of cancer metastasis from actual metastasis, meaning that in patients diagnosed with metastatic disease—and subsequently exposed to adverse-effect-evoking chemotherapy—the lymphatic system was in fact solely reactive to the tumor defense but does not represent lymph nodes with manifested metastasis. Biomarkers that can identify tumor malignancy such as predicting any possible elevated risk for the patient’s tumor to enter late stages of metastatic cascades are needed. Our results enforce a previously described collagen isoform to possess the potential to do so, meanwhile also opening a discussion to serve as a direct potential therapeutic target of colon cancer tumor microenvironment.

COL10A1 is a short-chain protein and member of the collagen family of proteins, which are major components of the interstitial extracellular matrix. In addition to the general structural functions of collagen, COL10A1 has also long been attributed to cell–cell interaction. Elevated expression levels have been observed in several malignant tumor types and correlate with tumor progression, invasion, metastasis, and vascularization (12). However, its role in CRC, particular in predicting tumor progression and tumor sub-stratification into cases that would benefit from neoadjuvant therapy, is insufficiently understood. Moreover, little information probing COL10A1 to serve as a micro-environmental niche factor that supports the progression of CRC is available.

## Material and methods

### Data obtain and preprocessing

Gene expression information and clinical factors of colon cancer were resourced from The Cancer Genome Atlas (TCGA) database. The count data needed to be transferred to transcripts per million (TPM) data format for the next step of the analysis. GSE14297, including 7 normal colon epithelium samples and 18 primary colorectal cancer tissues, were used to validate the gene expression difference between normal and tumor tissues. The cell line expression data was obtained from the Cancer Cell Line Encyclopedia (CCLE) database. Immune cell score data for each sample, according to gene expression, were conducted by ESTIMATE and immune-oncology biological research (IOBR) packages.

### Validation COL10A1 mRNA expression in tissues and cell lines

Colon cancer RNA-seq data from TCGA was used to conduct difference gene expression analysis to identify COL10A1 expression differences between normal and tumor tissues. Paired sample validation for COL10A1 was conducted by the TCGA data. In addition, COL10A1 expression difference was validated by the external GSE14297 dataset.

### Protein to protein interaction network calculation

Protein to protein interaction network is always used to identify a novel gene’s potential function and related network at the protein level. Here, we used the STRING database to show the interaction network of COL10A1 with STRING default setting. Cytoscape was used to visualize the final results.

### Association of COL10A1 activation with clinical variables

Patients’ clinical characteristics were extracted from the TCGA database. Two groups were formed, namely, one for baseline characteristics and another for tumor invasion factors, according to variable names. The next step was to analyze COL10A1 expression differences in different clinical features.

### Association of COL10A1 with consensus transcriptional markers defining tumor microenvironment

We calculated stromal, immune, and estimate scores for each patient based on COL10A1 expression, which was performed by ESTIMATE package. In addition, we also evaluated B cells, cancer-associated fibroblasts (CAFs), CD4 T cells, CD8 T cells, endothelial cells, macrophages, NK cells, and other cells infiltration scores for each sample using the IOBR package. In addition, we also explored this gene expression in single-cell level by an online tool (http://tisch.comp-genomics.org/home/). The detailed correlation between COL10A1 and immune cell markers was calculated by the Spearman test. Considering that immune checkpoints are important for tumor progression, exploring the relationship between COL10A1 and famous immune checkpoints (PD1, CD86, PDL1, CTLA4, LAG3, and TIM3) seemed to be necessary.

### Retrieval of COL10A1-related immunomodulators

TISIDB database (13) integrates the interaction between multiple immune genes and tumors. By entering the COL10A1 gene on the website and selecting samples of colon cancer, immunostimulatory factors and immune inhibitors significantly associated with COL10A1 expression can be calculated.

### Construction and validation of clinical prognosis signature

Immunostimulatory factors and immune inhibitors significantly associated with COL10A1 were selected from the original expression matrix. Then, we conducted univariate and multivariate Cox regression models to select candidate genes, which were combined with coefficient to construct a prognosis signature. The disease-free interval (DFI) was set as the outcome endpoint. Forty percent of the samples were randomly selected as a test dataset to validate the robustness of the model. This signature also was applied to test OS, PFS, and DSS.

### Protein extraction from cell cultures

Cells were lysed and harvested at >80% confluence in the culture flask using Cell Signaling Technology^®^ lysis buffer. The buffer was prepared, and 200 μl was added to a T-25 flask. Cells were scraped with cell scrapers and transferred to a Falcon tube. Subsequently, the cells were treated with ultrasound to ensure complete disruption. Insoluble cellular components in the lysate were separated by centrifugation (10 min at 14,000×*g*) in a 4°C tempered centrifuge. Supernatants were stored in aliquots at −80°C for further analysis.

### Protein extraction of CRC tissue samples

For suspension of the cell pellet, it was diluted 1:10 with radioimmunoprecipitation (RIPA) buffer. The buffer solution contained 10 ml RIPA buffer mixed with 50 μl phenylmethylsulfonyl fluoride (PMSF) and 100 μl protease inhibitor.

Cell lysis was performed using the FastPrep-24TM5G homogenizer. For this purpose, samples were transferred to 2-ml tubes containing Lysing Matrix E and homogenized three times for 30 s each at 8 m/s. For foam regression and final lysis, the samples were incubated on ice for additional 5 min. To separate the samples from the glass beads, a hole was pierced on the bottom of the matrix tube using a cannula. The tube was then placed in another 1.5-ml reaction tube and centrifuged at 3,000×*g* (3 min). The remaining insoluble components were removed by a second centrifugation step at 12,000×*g* for 5 min. The supernatant obtained was aliquoted and stored at −80°C.

### RNA isolation

Using the ReliaPrepTM miRNA Cell and Tissue Miniprep System, RNA was obtained directly from the culture flasks. At 90% confluence, the culture supernatant was removed; cells were washed with PBS and lysed using the kit’s lysis buffer and processed according to the manufacturer’s instructions. Subsequently, measurement of the RNA concentration and first quality control by photometric measurement with the NanoQuant PlateTM (Tecan) were performed.

### Isolation of recombinant COLX from overexpressing HEK2973-T

To obtain a positive control of ColX, the culture supernatant of cell line p52 (overexpressing recombinant COLX) was used. The p52 cells were inoculated into T-75 flasks. In these, the cells grew to a confluence of 80%. Then, media was changed from 5% to 0% fecal calf serum (FCS). After 72 h, the culture supernatant was removed.

The remaining cells were removed by centrifugation at 350×*g* for 5 min and transferred to a dialysis tube. Dialysis was performed for 24 h at 0.2 mM Tris, pH 7.5 with solution change after 8 h. Cells were removed by centrifugation at 350×*g* for 5 min. Meanwhile, water was changed twice. The dialysate was then transferred to glass flasks, frozen, and subsequently dried by lyophilization. The finished lyophilizate was then dissolved in water to achieve a 200-fold concentration of the culture supernatant. Since a yield of 50 μg/ml is expected, after lyophilization, an approximate final concentration of 10,000 μg/ml COLX is expected.

### Reverse transcription and qPCR

For reverse transcription, LunaScriptTM RT SuperMix Kit was used. One microgram of RNA was transcribed into cDNA for each sample, and a non-reverse transcriptase control was included for each sample for possible non-specific quantitative PCR (qPCR) reaction as caused by contamination with genomic DNA. After RT reaction, samples were diluted at 1:10.

The qPCR was also performed using the LunaScriptTM RT SuperMix kit, and samples were pipetted accordingly. The primers are shown in **Table 1**. All samples were plotted as triplets and analyzed as mean values. A non-reverse transcription control was also included from each sample to check for contamination with genomic DNA.

**Table 1:** Primers for qPCR of COL10A1 and GAPDH.

Gene	Orientation	Sequence from 5′ to 3′
**COL10A1**	F	AAA GGC CCA CTA CCC AAC AC
	R	ACC TTG CTC TCC TCT TAC TGC
**GAPDH**	F	CCT GTT CGA CAG TCA GCC GCA T
	R	GAC TCC GAC CTT CAC CTT CCC C

### Protein extraction, SDS-PAGE, and Western blotting

The protein concentration was determined using the Bradford assay with Bradford solution from Advanced Protein Assay Reagent (Cytoskeleton) kit. The absorbance was measured at 590 nm. A standard curve was generated using bovine serum albumin (BSA).

An adapted sodium dodecyl sulfate–polyacrylamide gel electrophoresis (SDS-PAGE) was performed to detect proteins of interest. The gel was loaded equally with 20 μg protein per gel well by measured protein concentration with Bradford assay for every sample guaranteeing a normalized and comparable standard for every sample. Proteins were denatured in advance with 4× Laemmli buffer + 8% mercaptoethanol at 95°C for 5 min. SDS-PAGE was run overnight in the refrigerator at 6.5 mA per gel.

The proteins separated by SDS-PAGE were transferred to a polyvinylidene difluoride (PVDV) membrane activated with methanol. The blotting chamber was filled with Towbin buffer, and blotting was performed for 90 min at 300 mA. After that, the blot was washed in 1× TBS buffer + 0.1% Tween 20 (TBS/T) for 15 min and then blocked in 5% milk powder (dissolved in TBS/T) for 60 min. After another 5-min wash step with TBS/T, incubation with the primary antibody (dissolved in 5% milk powder—TBS/T) was performed overnight. The next day, four washing steps with TBS/T followed once for 15 min and three times for 5 min. This was followed by incubation with the secondary antibody (dissolved in 5% milk powder—TBS/T) for 60 min. Subsequently, it was washed again four times with TBS/T (1× 15 min, 3× 5min). Finally, the ECL substrate was added to the blots. After 5-min incubation, the images were taken.

## Results

### COL10A1 transcripts are accumulated in CRC tissue samples but only in a subset of widely applied *in vitro* disease models

**Figure 1:**
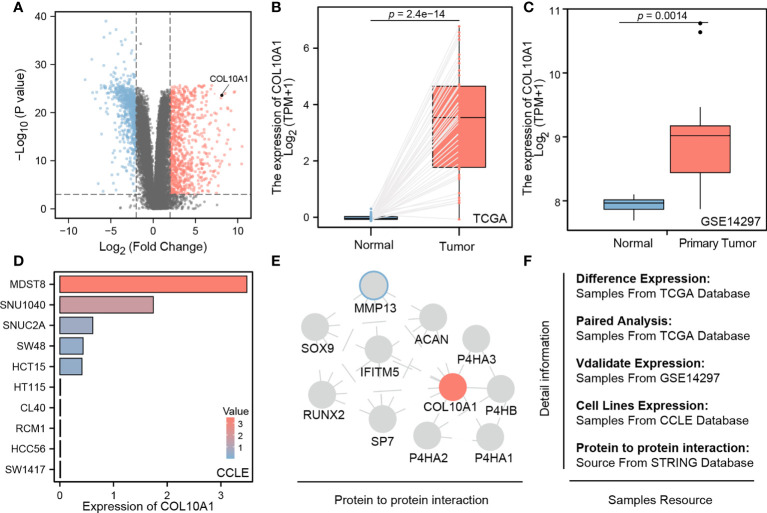
**(A)** COL10A1 upregulated mRNA levels in CRC tissues in comparison with all present genes; **(B, C)** paired sample expression validation of COL10A1 from TCGA and GSE14279; **(D)** different COL10A1 expression levels in common CRC cell lines; **(E)** microenvironmental protein–protein interaction of COL10A1; **(F)** data resources.

The differential expression results show that a total of 1,636 different genes, including 797 downregulated genes and 839 upregulated genes, were screened between normal and tumor tissues (**Table 2**). **Figure 1A** indicates that COL10A1 is significantly upregulated in CRC tissues, supported by paired-sample expression validation (**Figure 1B**) and external dataset GES14297 (**Figure 1C**).

**Table 2:** Multivariate Cox regression of COL10A1 related immunomodulators.

Gene symbol	coef	HR	95%CI (Low)	95%CI (High)	*p*-Value
CD244	−1.840308381	0.158768457	0.011655813	2.162648294	0.167248274
CD96	1.802432322	6.064380062	0.875891189	41.98775602	0.067889854
HHLA2	−0.459849416	0.631378714	0.447451581	0.890909984	0.008858794
PDCD1LG2	−1.574446309	0.207122203	0.043256938	0.991739331	0.048798872
TMIGD2	−1.242452254	0.288675444	0.055016077	1.514711989	0.141827337

To verify if widely distributed classical human *in vitro* models of CRC recapitulate physiological relevant levels of the gene, thereby enforcing their use in translational relevant research, we assessed datasets that retrieve expression data of standardized maintained cell lines. We found interesting differences between the cells models, as MDST8, SNU1040, SNUC2A, SW48, and HCT15 showed a significant upregulation of COL10A1. whereas HT115, CL40, RCM1, HCC56, and SW1417 had a very low-level expression (**Figure 1D**). Additionally, the STRING database shows that COL10A1 closely interacts with several proteins, of which some are famously described as potent promoters of cancer stem cells and mesenchymal transformation, such as MMP13, SOX9, and RUNX2. The computed interactome can be seen in **Figure 1E**. All the data resource has been shown in **Figure 1F**.

### Diversity-associated variances of COL10A1 expression

**Figure 2:**
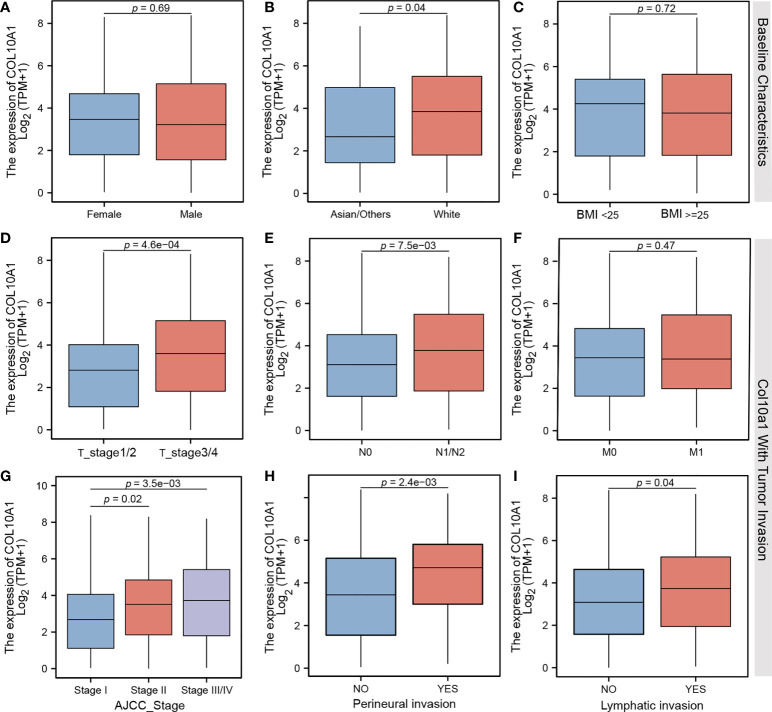
Comparison of different mRNA expression levels on **(A)** gender, **(B)** ethnicity, **(C)** body mass index, **(D)** TMN T stages, **(E)** TMN N stages, **(F)** TMN M stages, **(G)** AJCC Classification status, **(H)** perineural invasion, and **(I)** lymphatic invasion of CRC patients.

Although baseline characteristics are known to be determinants for clinical outcome, these characteristics such as sex, race, and body mass index (BMI) did not differ in their COL10A1 expression levels significantly. Nonetheless, Asians had a minimal higher expression level compared to other ethnicity (**Figures 2A–C**). To our knowledge, this is the hitherto first comparative assessment of COL10A1 appreciating different diversity setups.

### COL10A1 is increased in tumor with high invasion properties

To receive further insights in the clinical translation relevance of COL10A1 in CRC, we performed another screening of COL10A1 activation level with parameters typical describing advanced tumor cell invasion. A significant increase in COL10A1 expression was observed not only in stage T3 and T4 but also in N1 and N2 cases of CRC as compared to low-stage counterparts. Significant stratification did not occur in low-stage comparisons between T1, T2, and N0 (**Figures 2D, E**) nor was there a difference between M1 and M0 stages (**Figure 2F**). Elevated COL10A1 expression is associated with advanced tumor stages based on histopathological and image-based tumor staging. Moreover, tumors featuring perineural invasion—an established marker for predicting increased metastatic condition in CRC—have significant elevated levels of COL10A1 expression (**Figures 2G–I**).

### High COL10A1 levels are associated with elevated immune cell infiltration and extracellular matrix score

**Figure 3:**
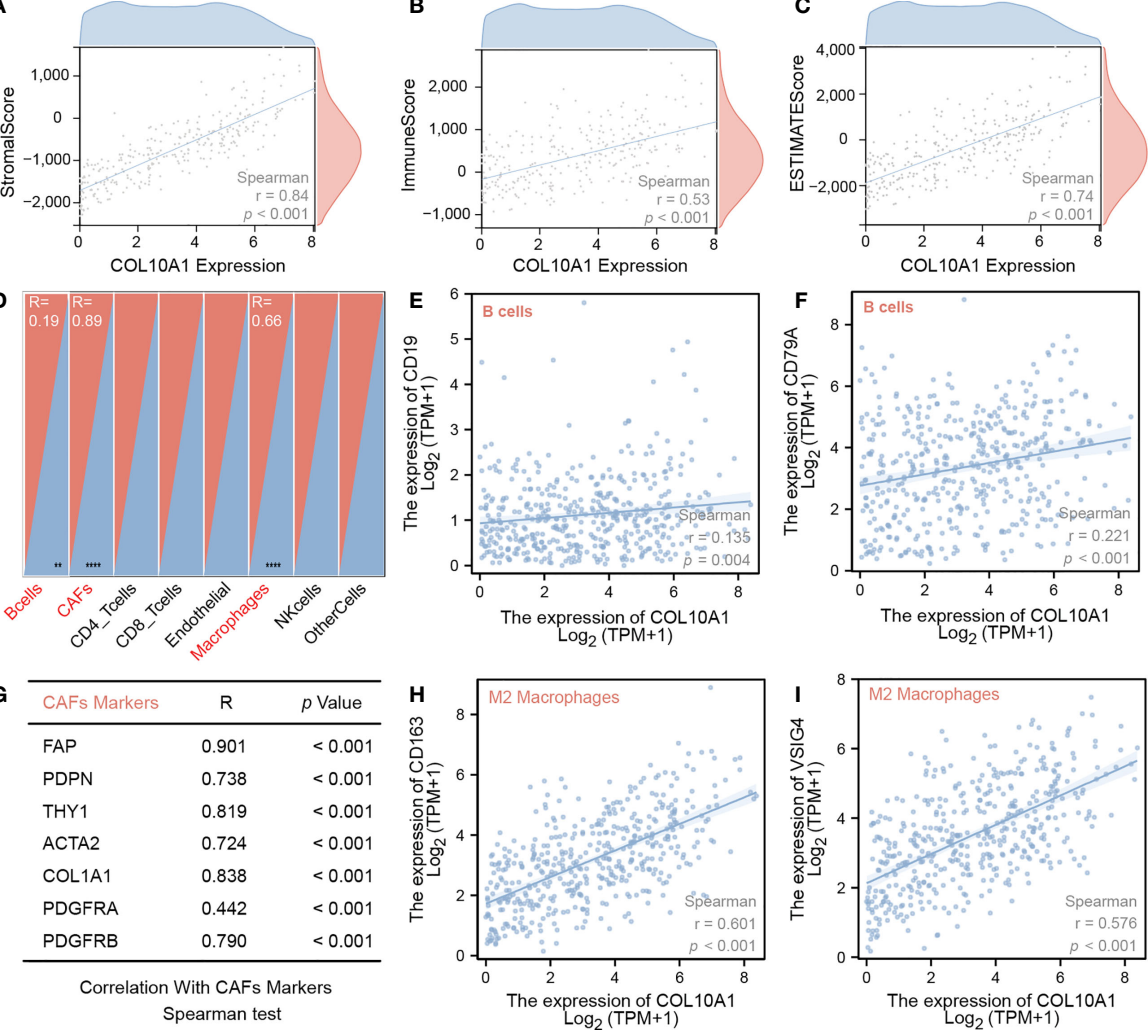
Spearman correlation test of COL10A1 mRNA levels and **(A)** stromal score, **(B)** immune score, **(C)** ESTIMATE score, **(E)** CD19, **(F)** CD79A, **(G)** different CAF cell markers, **(H)** CD163, and **(I)** VSIG4; **(D)** overview of correlation analysis of COL10A1 expression an several cell types.

**Figure 4:**
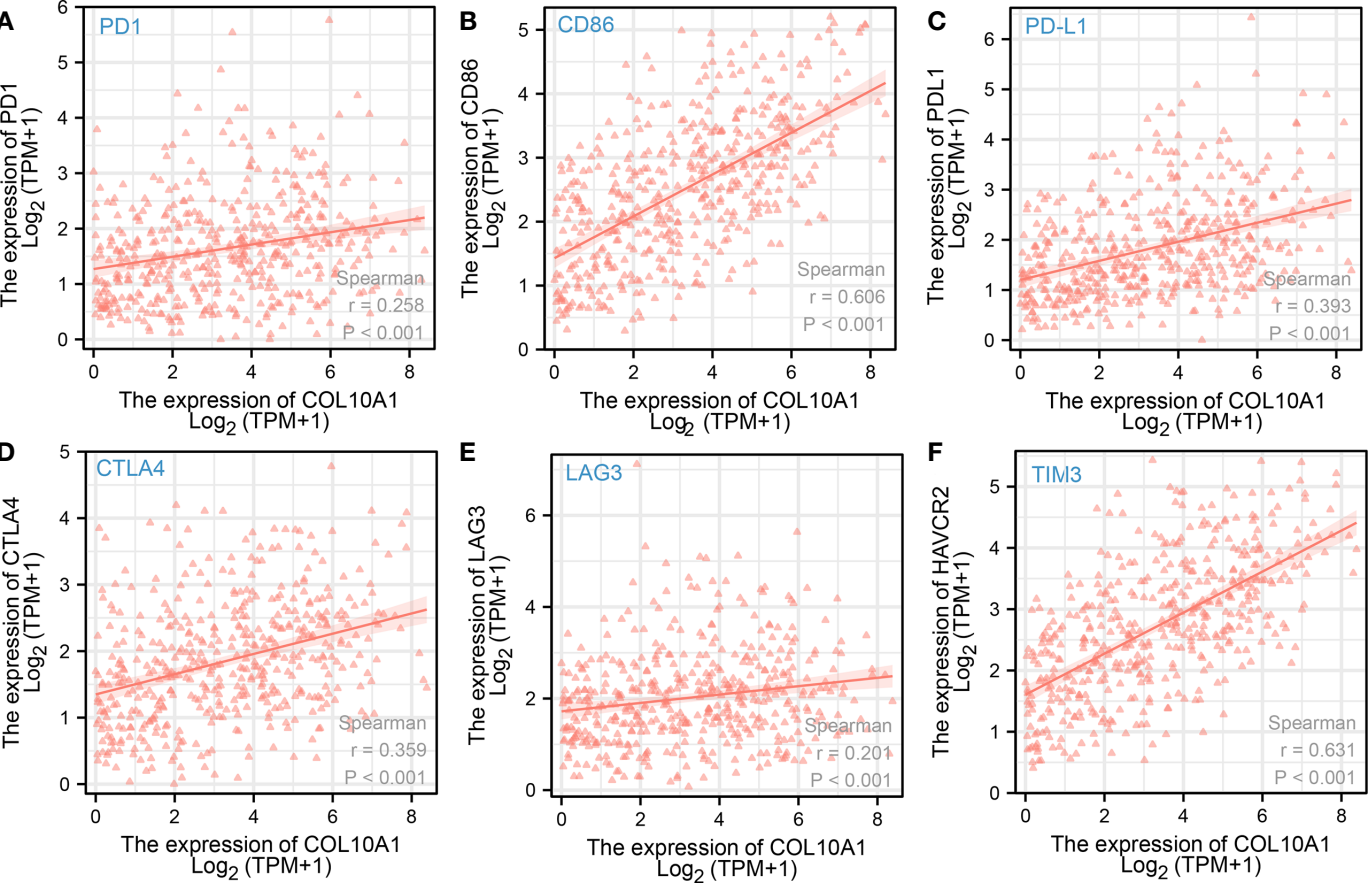
Spearman correlation test of COL10A1 expression levels and expression of immune checkpoint molecules **(A)** PD1, **(B)** CD86, **(C)** PD-L1, **(D)** CTLA-4, **(E)** LAG3, and **(F)** TIM3.

As a possible mechanism of how malignant cancers enforce their invasive and metastasis properties, the ability of cancer cells to modulate interactions with the immune microenvironment are discussed. In this line, we analyzed COL10A1 transcript in association with expression signals associated with the existence of immune cells and other parameters of immune cell infiltration. In addition to a clear positive correlation of increased COL10A1 expression with stromal immunity, we identified that the extracellular matrix score is upregulated in those cases (r=0.84, r=0.53, r=07, respectively; p<0.001, **Figures 3A–C**). Moreover, we found the COL10A1 cases are enriched of expression signals describing infiltration of B cells, CAFs, and macrophages (r=0.19, r=0.89, and r=0.66, respectively; p<0.001, **Figure 3D**). Further expression analysis confirmed these results, as we reveal a correlation of COL10A1 activation with respective consensus markers describing pools of cells such as B-cell markers (CD19, r=0.135, p=0.004; CD79A, r=0.221, p<0. 001), CAFs markers (FAP, PDPN, THY1, ACTA2, COL1A1, PDGFRA, and PDGFRB; p<0.001), and M2 macrophages markers (CD163, r=0.601, p<0.001; VSIG4 r=0.576, p<0.001) (**Figures 3E–I**). Moreover, the single-cell analysis results also demonstrate that COL10A1 could be expressed in CAF cells (**Supplementary Figure S1**). Of particular interest was also the significant correlation with immune checkpoint surface proteins such as PD1, CD86, PDL1, CTLA4, LAG3, and TIM3 (p<0.001) (**Figures 4A–F**), indicating a possible mechanism of how COL10A1-rich CRC facilitates invasion.

### COL10A1 related immunomodulators and construction of five gene risk signature to stratify patients’ survival probability

**Figure 5:**
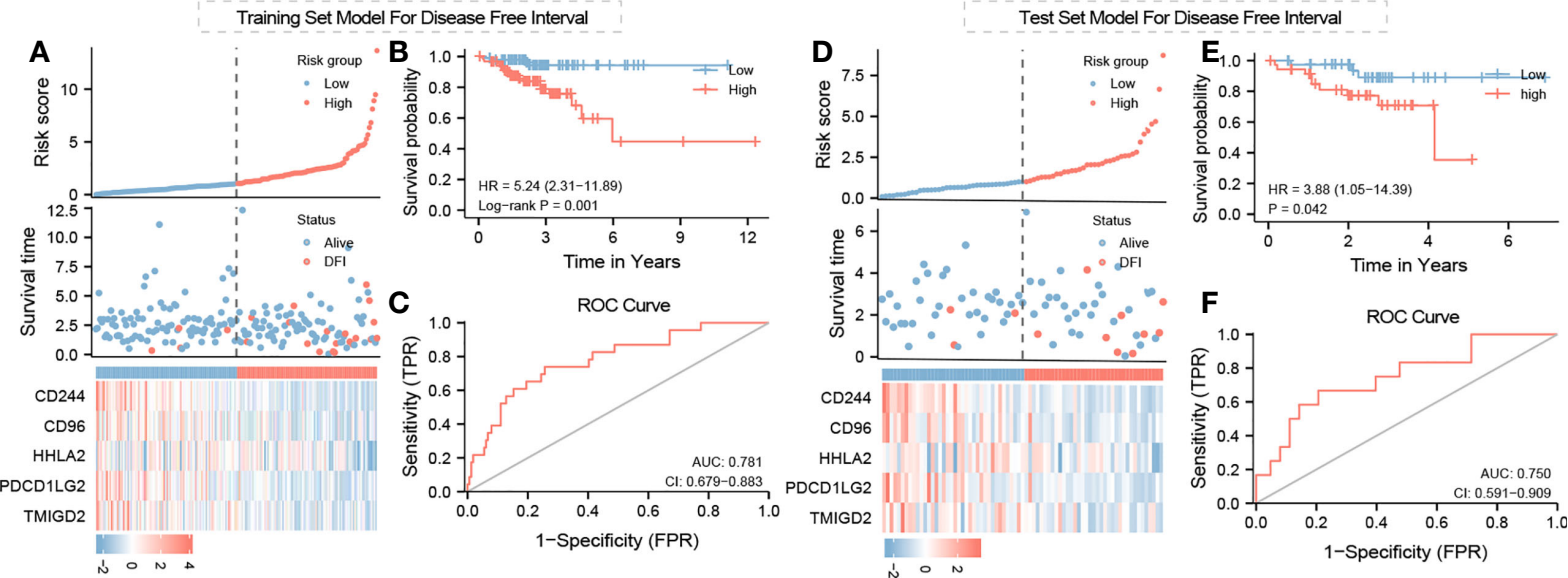
Training and test set model for DSS, PFS, and OS: risk score survival time and immunomodulator expression profiles regarding Col10A1 expression and risk group relation for **(A)** training set model and **(D)** test set model; **(B, E)** Kaplan–Meier curve for training set and test set model; **(C, F)** ROC curve for test model validation and optimization of training and test set model.

A total of 18 immunoinhibitors and 32 immunostimulators of COL10A1-expression-related immunomodulators were identified. Predictive model was built by Cox regression based on the above genes. Fourteen genes were demonstrated to affect patients’ outcome **(** **Supplementary Table S1** **)** by univariate results, and five genes are the main body of the model, which are inferred from multivariate results (**Table 2**). According to the median value of risk score, a high risk score means a poor outcome, while low-risk patients have a contrary prognosis. The area under the curve (AUC) was 0.781 **(** **Figures 5A–C** **)**, and the internal validation results also support the above conclusions, while the AUC was 0.750 in the validation dataset **(** **Figures 5D–F**). In addition, we applied the model to predict OS, PFS, and DSS, but the model did not accurately predict these new endpoints (**Supplementary Figure S2**).

### *In vivo* and *in vitro* analysis of COL10A1 expression levels in CRC

**Figure 6:**
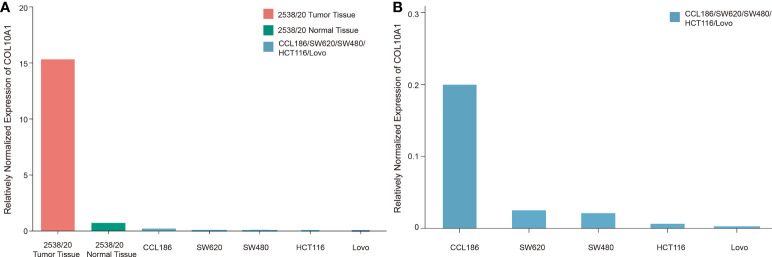
Relatively normalized expression (RNE) of COL10A1 in solid tumor, adjacent normal colon tissue, and CRC and fibroblast cell lines. For RNE, the sample with the lowest CT value is set as reference (value: 1) for other tissues and cell lines. CT values were normalized on GAPDH. **(A)** Illustration of multiple increased expression levels in CRC tumor stroma compared with *in vitro* cultured CRC cell lines and fibroblasts. **(B)** Enlarged breakdown of COL10A1 expression levels of common fibroblast and CRC cell lines. Fibroblasts (CCL186) were shown to have an eightfold increased expression compared to the strongest expressing tumor cell SW620.

Microarray analyses by Croner et al. (14) revealed significantly increased expression levels of COL10A1 in tumor tissue samples compared with that in normal tissues in CRC patients. Chapman et al. (15) successfully reproduced these data, but interestingly, high expression levels could not be found in *in vitro* cultured CRC cell lines. To address the question of whether tumor cells themselves exhibit increased expression of COL10A1, CT values were compared between tumor, normal tissue, fibroblasts, and CRC cell lines. This was accomplished by first normalizing the CT values of COL10A1 to GAPDH and second by comparing those RNEs (**Figures 6A, B**). The tumor tissue significantly shows the highest expression followed by the adjacent normal tissue, which is still higher than any expression of other cell populations, driving the hypothesis of COL10A1 overexpression in tumor stroma, triggered by lateral information transfer between tumor and stromal cells. Our hypothesis based on the bioinformatics analysis that CAFS/fibroblasts is the source of COL10A1 overexpression in CRC is strongly supported due to these results.

### COLX protein expression in several cancer and fibroblast cell lines

For PA-597603, the monomeric (∼75 kDa) and multimeric forms (∼140 kDa) were detected in our recombinant COLX from HEK2973 T cells. Bands at the level of the multimeric form were also weakly found in all CRC cell lines and in fibroblasts and skin sample. Particularly strong bands were detected in the range of ∼45 and ∼50 kDa and occasionally at ∼20 kDa.

The C-terminal antibody MA5-32504 was also able to detect the monomeric form of the recombinant protein at ∼75 kDa, which was absent in all other cell lines. In contrast, bands in the ∼66-kDa range and at ∼6.5 kDa (except LoVo and DLD-1) were detected here.

**Figure 7:**
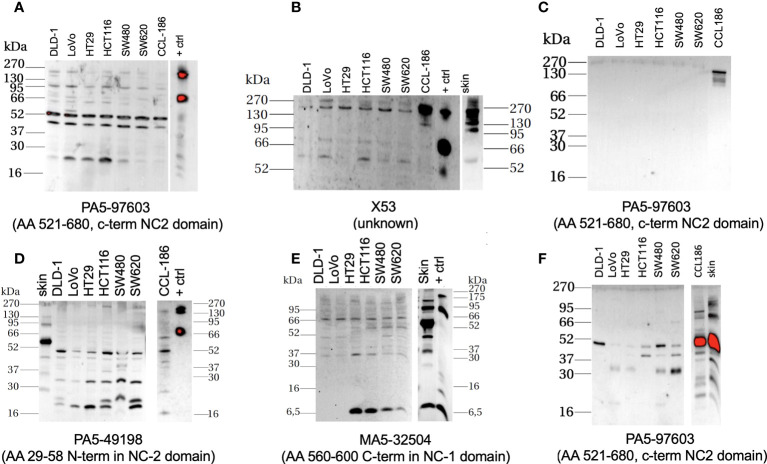
Western-blot-based assessment of COL10A1 with four different antibodies (antibody with its putative binding target labeled below the individual membranes, all Thermo Fisher) in extracts from classical CRC cell lines, fibroblast cell line, and skin. Little to low expression of the protein in cancer cells, elevated levels can be detected in the protein lysates and supernatants retrieved from fibroblasts/skin; positive control (+ ctrl) is purified recombinant protein from COL10A1 overexpressing HEK2973 T cells, housekeeping protein: GAPDH, antibody MA5-15738 (Thermo Fisher). **(A, B, D, E)** Cell lysates; **(C)** supernatants 20× concentrated by ultrafiltration, and **(F)** supernatants 70× concentrated by lyophilization.

The X53 antibody detected the monomeric form at ∼75 kDa and the multimeric form at ∼140 kDa in recombinant COLX. In cell lines, the multimeric form was detected at ∼170 kDa. The antibody PA5-49198 paralleled with PA5-97603, especially in all CRC cell lines, and in fibroblasts, the ∼50-kDa band was the most intense. Other bands were noted at ∼45, ∼32, and ∼20 kDa (**Figures 7A–F**).

## Discussion

Health issues associated with CRC is significant factor of the oncology-related health burden on the society. As prevalence directly relates to the socio-economic development of a country, besides the hotspots Western Europe, Australia, and North America, an increasing incidence can be observed in South America and Eastern Europe, mainly due to lifestyle changes, making CRCs as 1 out of 10 cancer cases in 2020 (CRC total, 1.9×10^6^, 935,000 deaths) (1), expected to reach 3×10^6^ incidences in 2040 (16). As prevalence rises, drug market size follows with an estimated size of 10.9 billion dollars in 2022 (17) in five most prevalent countries, mainly attributed to adjuvant and neoadjuvant chemotherapy of surgically resectable CRC cancers.

Surgery is the main type of treatment with the strongest positive clinical prognostic consequences for all CRC combined, featuring a rising cost of surgery in recent decades (30,000 cases in 2015 in Germany, CRCs cost of illness ratio reaching roughly 50,000 EUR per patient). The surgical treatment procedure for confirmed CRC depends significantly on whether existing metastasis locally in surrounding lymph nodes exists or not, defining applied resection technique and resection size and aggressive adjuvant therapy. Thus, an accurate diagnosis of this status has an immense impact on the operation procedure and the patient’s rehabilitation after surgery. The procedure depends mainly on the localization of the arterial transection, which determines the size of resected colon/rectum segments. Thus, a lymphogenic metastasis follows the regular pattern of an initially longitudinal paracolic (maximum of 10 cm) and then truncal metastasis, which then decides the intestinal resection size with possible partial resection of infiltrated neighboring structures. In colon carcinoma, the spectrum ranges from segmental or hemicolectomies up to complete mesocolic excision (CME) depending on infiltration depth and N status. This will also determine the level of lymphatic resection, i.e., D2—paracolic and intermediate lymph nodes or D3—main, paracolic and intermediate lymph nodes, which is comparable to western CME (18). Therefore, it is becoming increasingly important to improve diagnostic accuracy. A biomarker in the primary tumor that trustfully predicts actual tumor spread would impact both types of the treatment decision. Our project associates to this initiative.

Previous works of others have identified some promising biomarkers; however, hitherto, there is no consensus marker established to support clinical decision making in the before-mentioned manner. Given the economic and clinical importance, the field of research is relatively large and allows only an insufficient discussion of the development. The most prominent example has been initially discovered about a decade ago: Stein et al. (19) identified the gene metastasis-associated in colon cancer 1 (MACC1) to possess strong predictive potential to distinguish CRC metastasis risk, and the abundance of the related DNA string in the blood of patients can help to identify cancer recurrence and therapy response. The diagnostic value has been described in independent clinical cohorts, however mostly fundament on retrospective trials (20). To our knowledge, no association to surgical decision making or omission of neoadjuvant treatment in respective MACC1 low-expressing patients in a prospective manner has been conducted. It would be interesting to study the correlation of MACC1 and COL10A1 activation in the analysis of bulk tumor specimen and in functional studies applying genetic COL10A loss of function models. From the similar historic time span, in the early 2010, Smith et al. constructed a 34-gene signature that predicts the metastatic spread of CRC based on the experimental model of tumor metastasis (21). Although their signature was validated in prospective clinical trials, Smith signature has not been established in clinical routine, probably due to the necessity of conducting at least 34 multiplex analytics hindering simple and rapid dissemination as POCT. Interestingly, a group at Fudan University analyzed parts of the same datasets that we assessed in this project and identified a five-gene signature that predicts metastasis spread (22). COL10A1 did not come up as their top candidate suggestion, urging that interrogation of the Fudan signature in the context of COL10A1-rich CRC is needed. Very recently, Liu et al. proposed a hub gene signature comprised of four candidates, and the corresponding protein accumulation in the tumor material was verified in independent prospective cohort of patients (23). Importantly, as the new research field of cancer neuroscience currently emerges, indication from the experimental field emerges that nerve growth factors mediate liver metastatic potency of CRC cells (24). Further studies to decipher the composition and tumor-relevant roles of the neural microenvironment at the primary tumor site or in the metastasis site of intestinal tumors are needed and surely will reveal new insights in the diseases. As such, our group recently identified the hitherto unrecognized clinical prognostic role activation levels of sensory nerve chancel of the transient receptor potential channel (TRPC) class in pancreatic cancer (25), a discovery relevant also for developing new therapies for this deadly disease, as members of TRPC are druggable targets with clinical applied inhibitors.

We acknowledge that our work is of a descriptive nature only, and our assumptions are made based on correlative findings. We consider our results to be relevant for the field: our study relies on reusing various publically available, high-quality molecular data from larger patient population retrieved from different datasets that all have been quality approved by the scientific community. It reflects and discriminates regarding gender and ethnic diversity. It is based on current sequencing technologies and molecular tumor diagnostic data, and in our data analysis, we appreciate the importance of the emerging field of intra-tumor spatial heterogeneity to instruct the biological behavior of the disease. As COL10A1-enriched tumors feature increased immune cell infiltration and extracellular matrix components, we assume that CRC COL10A1 activation might either modulate the tumor microenvironment, or vice versa; its expression is a downstream signal of altered immune and stromal environmental interactions. Of particular interest is the very strong correlation of elevated COL10A1 transcription with CAFs, as those pool of cell populations are emerging as modulators of establishing a pro-invasive tumor microenvironment. Functional studies to address this questions, particularly using human model systems that recapitulate cellular and spatial heterogeneity as achieved in patient-derived organoids (26), are underway in our lab. Of note, all of our lab-tested classical CRC cell models show a low abundance of COL10A1 protein expression in **Figure 7**, which in part reflects the results of the cell line transcription data. However, in our view, it urges the assessment of COL10A1 in clinically more relevant 3D model systems featuring the stroma microenvironment (27). It will be interesting to compare the mRNA/protein levels of COL10A1 in the primary tissue with matching personalized 3D *in vitro* models and study effects of COL10a1 modulation in such conditions. Of note, although not retrieved from orthotopic condition and also resembling high *in vitro* passage model, the tested fibroblasts are high in COL10A protein. Confirmatory studies in patient-matched tumor cell/CAFs co-culture systems are needed to analyze COL10A1 protein/DNA as component of the lateral information system between tumor cells and stroma environment.

The described results further established COL10A1 as a diagnostic marker for predicting progression of colon carcinogenesis, extending previous reports on this protein in the context of colon cancer. The first mentioning of COL10A1 to be specifically upregulated in CRC as compared to normal mucosa related back to Croner et al. in the year 2005 (14). After that, the notable report by Huang et al. described the upregulation of COL10A1 compared to the control tissue in 30 patients (28). Moreover, using protein-based quantification of COL10A1 in tumor specimens based on histological staining and semi-quantitative signal quantification in 197 CRC patients, they identified the significant clinical negative prognostic value of an elevation of the biomarker. Furthermore, a historic study has already proposed COL10A1 serum protein levels to be a minimally invasive and indicative marker for colon cancer detection as compared to its absence in healthy patients (12). It would be interesting to investigate if blood serum levels of COL10A1 protein share a similar prognostic value regarding the metastatic spread of the primary disease as compared to its mRNA abundance in tumor specimens, and to perform a confirmatory study on tumor detection like that reported by Solé et al. In addition, using machine learning algorithm and advance materials to discover the potential value of these gene is also a promising research topic (29, 30). Our data support the initiation of a relevant prospective clinical study to assess COL10A1 expression in tumors aiming to improve the management of colon cancer patients with enlarged lymph node, either by stratifying patient cohorts who do not need to receive neoadjuvant chemotherapy or minimizing the number of patients that require more comprehensive surgical attempt of D3 lymph node resection.

## Data availability statement

The datasets presented in this study can be found in online repositories. The names of the repository/repositories and accession number(s) can be found in the article/**Supplementary Material**.

## Author contributions

RC, AP, and UK: Conceptualization, data curation, formal analysis, roles/writing—original draft, writing—review and editing. RC, UK, WS, MS, LS, TW, MD, MM, and AB: Roles/writing—original draft. RC, AP, and UK: Funding acquisition, methodology, project administration, resources, supervision. All authors contributed to the article and approved the submitted version.

## Acknowledgments

UK thanks M Stuerzl, University of Erlangen for stimulating discussion on the phone.

## Conflict of interest

The authors declare that the research was conducted in the absence of any commercial or financial relationships that could be construed as a potential conflict of interest.

## Publisher’s note

All claims expressed in this article are solely those of the authors and do not necessarily represent those of their affiliated organizations, or those of the publisher, the editors and the reviewers. Any product that may be evaluated in this article, or claim that may be made by its manufacturer, is not guaranteed or endorsed by the publisher.

## Supplementary material

Supplementary Figure 1 COL10A1 expression levels. COL10A1 expression is high in CAF cell cluster **(A, B)** as well as in fibroblasts **(C)**.

Supplementary Figure 2 Kaplan-Meier curves of COL10A1 expression levels in groups with „high” and „low” expression on OS **(A)**, PFI **(C)**, DSS **(E)**, ROC curve for validation of Kaplan-Meier curves for OS **(B)**, PFI **(D)**, DSS **(F)**.

## Abbreviations

CRC, colorectal cancer; COL10A1, collagen type X alpha 1; COLX, Collagen X; CAF, cancer-associated fibroblast; DRE, digital rectal examination; CEA, carcinoembryonic antigen; OS, overall survival; DFS, disease-free survival; DSS, disease-specific survival; TCGA, The Cancer Genome Atlas; TPM, transcripts per million; CCLE, Cell Line Encyclopedia; DFI, disease-free interval; GAPDH, glycerinealdehyde-3-phosphate dehydrogenase; CME, complete mesocolic excision; MACC1, metastasis-associated in colon cancer 1; TRPC, transient receptor potential channel; CTC, CT colonography; DRE, digital rectal examination; IOBR, immune-oncology biological research.
