# Supplementary figures and images for: COL10A1 allows stratification of invasiveness of colon cancer and associates to extracellular matrix and immune cell enrichment in the tumor parenchyma
Source: Front Oncol. 2022 Oct 4;12:1007514. doi: 10.3389/fonc.2022.1007514 (PMC9577326; doi:10.3389/fonc.2022.1007514)

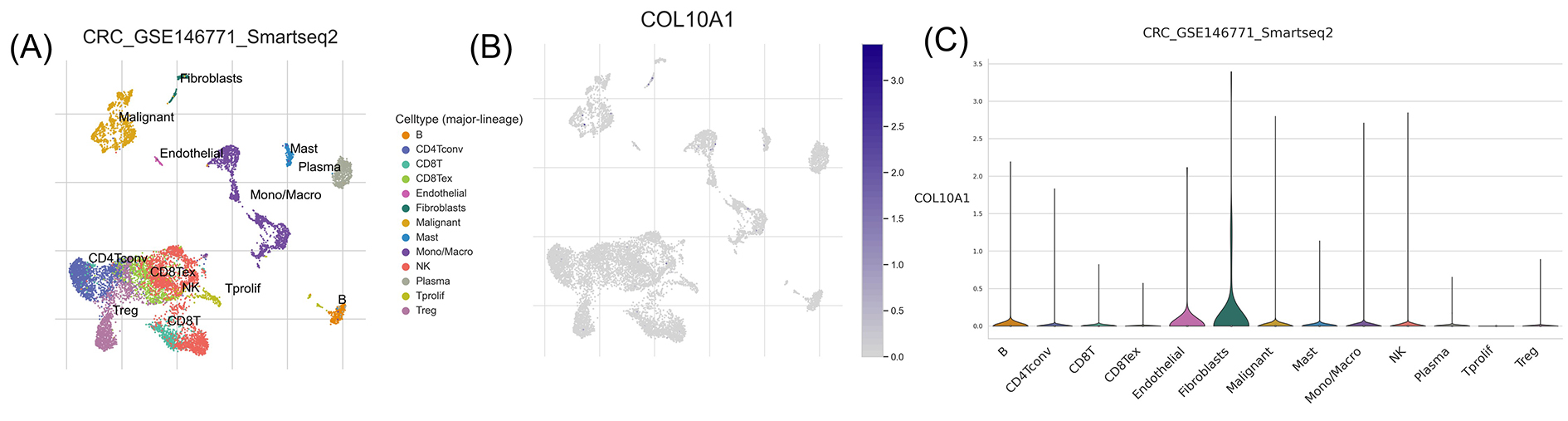

Supplement: Supplementary Figure 1 — COL10A1 expression levels. COL10A1 expression is high in CAF cell cluster (A, B) as well as in fibroblasts (C). [file Image_1.jpeg]

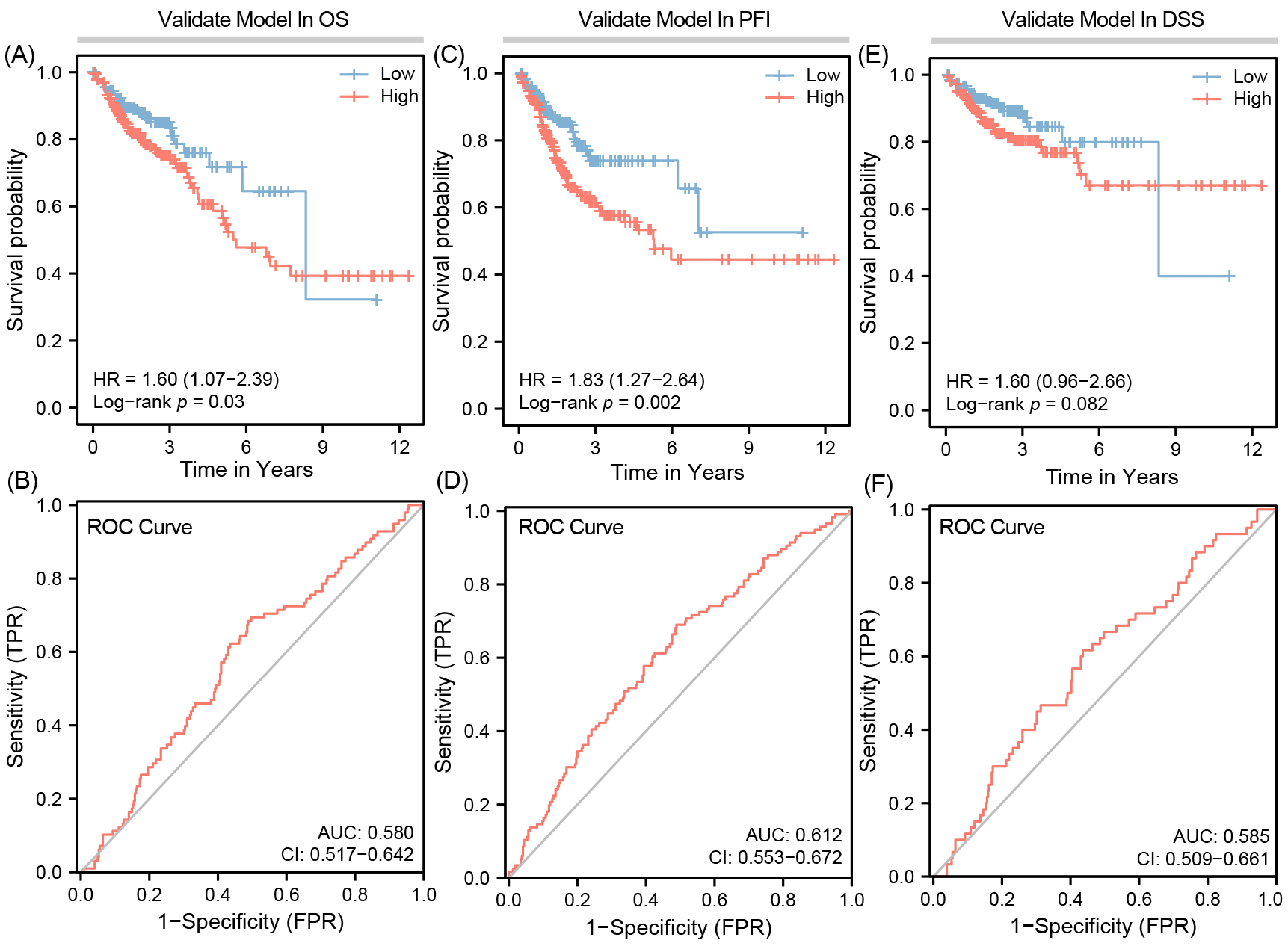

Supplement: Supplementary Figure 2 — Kaplan-Meier curves of COL10A1 expression levels in groups with „high” and „low” expression on OS (A), PFI (C), DSS (E), ROC curve for validation of Kaplan-Meier curves for OS (B), PFI (D), DSS (F). [file Image_2.tif]
